# Human Multi-Compartment *Airways-on-Chip* Platform for Emulating Respiratory Airborne Transmission: From Nose to Pulmonary Acini

**DOI:** 10.3389/fphys.2022.853317

**Published:** 2022-03-08

**Authors:** Eliram Nof, Hikaia Zidan, Arbel Artzy-Schnirman, Odelia Mouhadeb, Margarita Beckerman, Saurabh Bhardwaj, Shani Elias-Kirma, Didi Gur, Adi Beth-Din, Shulamit Levenberg, Netanel Korin, Arie Ordentlich, Josué Sznitman

**Affiliations:** ^1^Department of Biomedical Engineering, Technion—Israel Institute of Technology, Haifa, Israel; ^2^Israel Institute for Biological Research, Ness Ziona, Israel

**Keywords:** lungs, *organ-on-chip*, *in vitro*, preclinical models, inhalation, respiratory disease, microfluidics, SARS-CoV-2

## Abstract

The past decade has witnessed tremendous endeavors to deliver novel preclinical *in vitro* lung models for pulmonary research endpoints, including foremost with the advent of *organ-* and *lung-on-chips*. With growing interest in aerosol transmission and infection of respiratory viruses within a host, most notably the SARS-CoV-2 virus amidst the global COVID-19 pandemic, the importance of crosstalk between the different lung regions (i.e., extra-thoracic, conductive and respiratory), with distinct cellular makeups and physiology, are acknowledged to play an important role in the progression of the disease from the initial onset of infection. In the present Methods article, we designed and fabricated to the best of our knowledge the first multi-compartment human *airway-on-chip* platform to serve as a preclinical *in vitro* benchmark underlining regional lung crosstalk for viral infection pathways. Combining microfabrication and 3D printing techniques, our platform mimics key elements of the respiratory system spanning (i) nasal passages that serve as the alleged origin of infections, (ii) the mid-bronchial airway region and (iii) the deep acinar region, distinct with alveolated airways. Crosstalk between the three components was exemplified in various assays. First, viral-load (including SARS-CoV-2) injected into the apical partition of the nasal compartment was detected in distal bronchial and acinar components upon applying physiological airflow across the connected compartment models. Secondly, nebulized viral-like dsRNA, poly I:C aerosols were administered to the nasal apical compartment, transmitted to downstream compartments via respiratory airflows and leading to an elevation in inflammatory cytokine levels secreted by distinct epithelial cells in each respective compartment. Overall, our assays establish an *in vitro* methodology that supports the hypothesis for viral-laden airflow mediated transmission through the respiratory system cellular landscape. With a keen eye for broader end user applications, we share detailed methodologies for fabricating, assembling, calibrating, and using our multi-compartment platform, including open-source fabrication files. Our platform serves as an early proof-of-concept that can be readily designed and adapted to specific preclinical pulmonary research endpoints.

## Introduction

The past decade has seen tremendous endeavors to deliver novel preclinical *in vitro* lung models for pulmonary research endpoints (Sakagami, 2006, 2020; Hittinger et al., 2015; Ehrmann et al., 2020; Selo et al., 2021). The motivation for such human-relevant *in vitro* respiratory models is multifold but has been significantly thrusted by efforts to circumvent critical hurdles prevalent in *in vivo* animal experiments. Notably, animal models differ by important underlying discrepancies with humans, spanning amongst other anatomical and physiological differences between species (Hogg and Timens, 2009) to broad divergences in immunological (Mestas and Hughes, 2004) and genetic (Seok et al., 2013) responses to inflammatory diseases. Not only do these dissimilarities translate to contrasting delivery protocols when considering *in vivo* animal experiments (Wylie et al., 2018), but the translational impact of *in vivo* findings remains frequently questioned in characterizing human diseases (van der Worp et al., 2010). Most significantly, the gap between humans and animals constitutes an inevitable barrier to new therapeutic development (Barnes et al., 2015a; Prakash et al., 2017) and is underscored with as high as 80% failure on drug efficacy in human trials leveraging molecules previously screened in rodent lungs (Miller and Spence, 2017). This reality is of important concern as respiratory diseases represent a growing worldwide healthcare burden associated with high morbidity and mortality (Barnes et al., 2015b; Wisnivesky and De-Torres, 2019); meanwhile, respiratory therapies have seen much fewer drugs approved in past decades than other areas of medicine (Barnes et al., 2015a) (e.g., cardiovascular, neurology).

In light of ongoing *in vivo* limitations, *in vitro* cell-based assays have served as useful preclinical “gold standards” in respiratory research. This has included transwell inserts, where human-relevant cell cultures are grown on porous membranes that recapitulate the luminal airway barrier characteristics and, crucially, the air-liquid interface (ALI) (Forbes and Ehrhardt, 2005; Nahar et al., 2013; de Souza Carvalho et al., 2014; Nichols et al., 2014; Faber and McCullough, 2018; Lacroix et al., 2018). Yet, the most exciting progress has arguably arisen from the field of microfluidic *lung*- and *organ-on-chips*. Unlike traditional *in vitro* setups, *airway-on-chips* enable the exchange and collection of media (e.g., analytics of inflammation) with the integration of continuous perfusion from either the basal (i.e., fluid) and/or apical (i.e., air) side of a porous membrane (Tenenbaum-Katan et al., 2018). In recent years, microfluidic platforms that recapitulate more intimately physiological and biological functions have opened new opportunities for human disease modeling, drug discovery and screening, as well as other translational applications (Bhatia and Ingber, 2014; Benam et al., 2015; Esch et al., 2015; Liu et al., 2017; Zhang et al., 2018; Haddrick and Simpson, 2019; Mittal et al., 2019; Shrestha et al., 2020; Artzy-Schnirman et al., 2021; Ma et al., 2021). In turn, various commercial *lung-on-chip* models (Ainslie et al., 2019) are now widely available to the scientific and biopharmaceutical community. In parallel recent microfluidic open-source designs of perfused ALI models (Carius et al., 2021) have been made accessible toward broader and simpler end-user applicability (Artzy-Schnirman et al., 2019a). Altogether, the growing prevalence of these human-relevant *organ-on-chips* has opened new debates on whether preclinical *in vitro* research has reached maturity in so far as to bypass *in vivo* animal validation studies (Ingber, 2020).

Despite the aforementioned advances, *lung-on-chips* are still overwhelmingly limited to models of single channels or individual alveolar-like cavities that often forfeit anatomical traits of the respiratory organ and corresponding respiratory airflow physiology characteristics (Tenenbaum-Katan et al., 2018; Artzy-Schnirman et al., 2021). Most recently, *in vitro* cytotoxicity and inflammatory assays have highlighted more realistic microfabricated tree-like anatomies of either bronchial (Elias-Kirma et al., 2020) or alveolar (Artzy-Schnirman et al., 2019b) airway networks. Nevertheless, by and large virtually all existing microfluidic *in vitro* lung models still focus on isolated airway assays that discard the anatomical continuity between the distinct regions of the lungs, i.e., spanning the extra-thoracic (i.e., nose-throat), conductive (i.e., bronchial) and respiratory (i.e., alveolar) airways, and ensuing crosstalk that can arise *in vivo* between one another. This latter aspect becomes critical when addressing for example the fate of inhaled aerosols and the so-called “journey” of airborne particles along the respiratory tract (Artzy-Schnirman et al., 2020); an area that has drawn considerable interest from numerical *in silico* modeling efforts (Koullapis et al., 2019), most notably with computational fluid-particle dynamic (CFPD) simulations of respiratory airflows and aerosol transport, but is otherwise notoriously challenging to mimic *in vitro* (Artzy-Schnirman et al., 2019a).

The call for novel *in vitro* solutions has been further strengthened amid the ongoing coronavirus disease 2019 (COVID-19) pandemic in deciphering the determinants governing the aerosol transmission of respiratory viruses (Leung, 2021; Wang et al., 2021). It is now established that infection with the severe acute respiratory syndrome-related coronavirus 2 (SARS-CoV-2) virus typically initiates in the upper respiratory tract (e.g., nasal-oral cavities), highlighting the nasal susceptibility to SARS-CoV-2 due to higher presence of angiotensin-converting enzyme 2 (ACE2) expression in the nasal epithelium with decreasing expression throughout the lower respiratory tract (Hou et al., 2020). Yet, severe symptoms of the disease are acknowledged to arise from infection and associated inflammation of alveolar epithelial cells in the distal lungs (Borczuk et al., 2020; Tay et al., 2020). While infection could originally initiate in the deep lungs via inhalation of fine virus-laden aerosols that may directly deposit on the alveolar surfaces (Wang et al., 2021), it has been hypothesized that deep lung viral infection likely ensues from subsequent aspirations following early infection in the naso-oropharyngeal region (Hou et al., 2020). In the absence of definitive evidence, advanced human-relevant *in vitro* pulmonary models that recapitulate key aspects of the different lung regions could offer attractive opportunities to shed new light on the mechanistic determinants at the origin of the initial onset of respiratory infections in the distal lungs.

**FIGURE 1:**
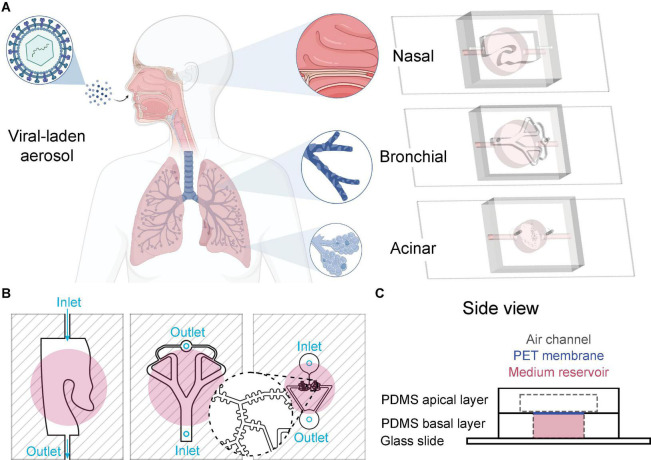
Multi-compartment *lung-on-chip* platform. **(A)** The *in vitro* multi-compartment platform recapitulates three key elements of the flow physiology and cellular makeup across the respiratory tract: (i) nasal passages, (ii) bronchial airways, and (iii) acinar airways, respectively. The platform’s “plug and play” modularity may be leveraged toward various benchmark *in vitro* assays for human host-pathogen interaction studies, including investigations of viral airborne transmission. **(B)** Schematic of the three individual chips (top view). From left to right: nasal, bronchial, and acinar airways, respectively. Inlets and outlets of apical chambers are designated with light blue, and reservoir chambers marked with pink circles (reservoir inlets and outlets are not shown in the figure). **(C)** Example of the side view for the nasal chip, composed of (1) an apical PDMS layer that captures relevant nasal passages (see top view in **B**); (2) a polyester-based (PET) porous membrane between apical and basal layers, where epithelial cells are grown; (3) a basal PDMS layer containing a fluidic reservoir to supply media and essential nutrients needed for cell culture.

Motivated by these broad questions, we introduce in the present Methods article, to the best of our knowledge, the first multi-compartment airway-on-chip model recapitulating key anatomical and physiological components of the respiratory regions. Our versatile platform (see Figure 1A) encompasses three distinct chips mimicking, respectively, (i) nasal passages, (ii) mid-bronchial airways, and (iii) distal alveolated airways reminiscent of the pulmonary acinus. First, we detail the engineering methods to microfabricate each distinct fluidic compartment (i.e., nasal, bronchial and acinar) that may be sequentially inter-connected to recreate crosstalk via representative inhalation airflows. We next describe detailed protocols to recreate human-relevant epithelial airways at the ALI, pertinent to the cellular makeup of each respective lung region. As a proof-of-concept of the multi-compartment’s usability, we explore *in vitro* determinants of aerosol transmission of respiratory viruses in several exemplary assays. To this end, we isolate the nasal chip from the other compartments and expose its epithelium with an instilled viral suspension. Following infection of the nasal compartment as the alleged site of initial COVID-19 infection in the lungs, bronchial and alveolar compartments are then connected in series to the nasal chip and subjected to inhalation airflows spanning the nose to acini. We subsequently observe infection in both distal epithelial compartments mediated via airflows. Finally, we showcase the initial onset of an inflammatory response across the respective airway barriers in each compartment upon inhalation of nebulized viral-like dsRNA laden aerosols. Together, these preliminary *in vitro* assays support the hypothesis of subsequent aspiration as a potent mechanism for viral transmission across the broader respiratory landscape. Overall, the Methods presented herein underline the applicability of the multi-compartment microfluidic platform toward a broad array of preclinical *in vitro* respiratory research endpoints, where airflow crosstalk and airborne transmission mechanisms are presumed central.

## Materials and Methods

### Device Design

The respiratory tract is an intrinsically complex and multi-scale organ that exhibits a vast network of anatomical structures with a distinct cellular makeup spread over length scales spanning several orders of magnitude (Tenenbaum-Katan et al., 2018; Artzy-Schnirman et al., 2021). By definition, all *in vitro* models are limited in mimicking some but not all characteristics of the *in vivo* environment; a realism that has been recently discussed in some depth (Artzy-Schnirman et al., 2021). As with all *in vitro* pulmonary assays, our designs do not claim to recreate the entire airway pathlength from extra-thoracic regions (i.e., head) to the pulmonary acini. Rather, our specific endpoints lie in capturing some key anatomical and cellular features of the lungs’ relevant airway regions; a strategy in line with delivering human-advanced preclinical *in vitro* solutions to elucidate critical aspects relevant to the airborne journey and aerosol transmission phenomenon across the lungs (Artzy-Schnirman et al., 2019a). To this end, we select the nasal passages, bronchial airways and acinar regions as three compartments representative of the extra-thoracic, conductive and respiratory regions, respectively (see Figure 1A). Here, specifically, the apical partition of each chip compartment is designed to recapitulate relevant geometrical features of each lung region, and most critically where anatomical structures are known to influence airflow mechanics (Sznitman, 2021). Below we detail design considerations relevant to each compartment.

#### Nasal Compartment Design

**FIGURE 2:**
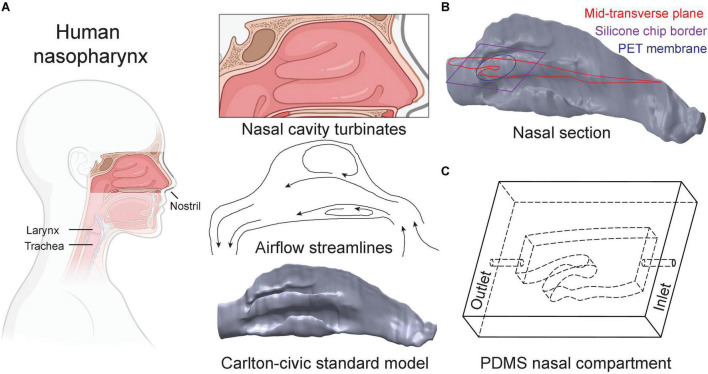
Design of the nasal compartment chip. **(A)** The nasal cavity features a complex network of turbinate airway passages. The Carlton-Civic Standard Nasal model serves as the basis for our nasal chip design. Nasal flows are dominated by the turbinate structures giving rise to recirculating zones, as illustrated schematically by airflow streamlines in the nasal passages. **(B)** We recapitulate elements of nasal flow characteristics in a PDMS compartment based on the generic nasal model. A cross-section of the selected geometry is defined parallel to the bulk flow direction (i.e., beginning upwards through the nostril but then curves toward the elongated axis of the trachea) and bound to a maximal size fitting a standard glass microscopy slide (i.e., 40 mm). **(C)** Schematic drawing depicts the resulting nasal chip compartment, with inner cavities marked by dashed lines and labeled “inlet” and “outlet,” respectively, where holes are punched into the PDMS material to connect tubing (see text for details).

**FIGURE 3:**
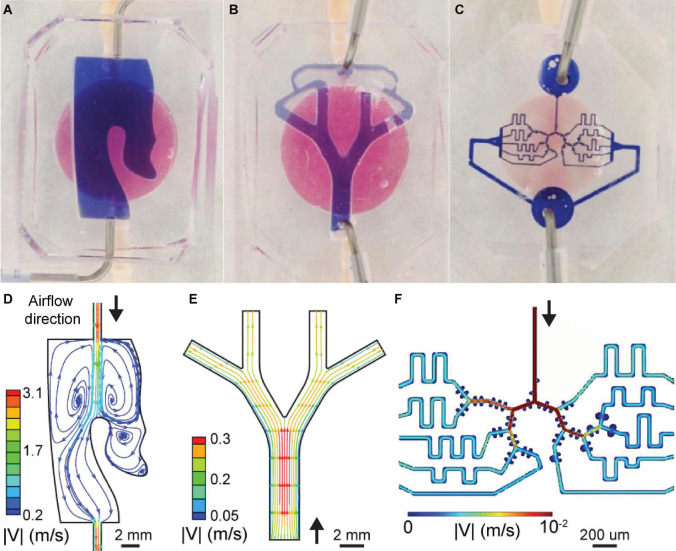
Characteristic flow features in the multi-compartment airway-on-chip platform. **(A–C)** View of the inter-connected chip compartments, where the reservoir, i.e., basal chamber, filled with pink media, and the geometry channel, i.e., apical chamber, filled with blue media are shown for imaging contrast only. **(D–F)** Steady-state flow patterns are resolved in each of the three compartments using CFD simulations and visualized by plotting streamlines colored according to velocity magnitude (see text for detail). Each compartment is subject to physiologically relevant flows (see Table 1), highlighting order of magnitude changes in the velocity scales between each lung compartment, reminiscent of physiological pulmonary fluid dynamics. **(D)** Flow in the nasal compartment is characterized by recirculation zones attributed to the turbinate pocket structure. **(E)** Poiseuille-like flows develop at the inlet of the bronchial model, subsequently splitting and weakened as is typical of flow in a symmetrically bifurcating bronchial airway geometry. **(F)** Flow velocity contours in the acinar model, characterized by generation-level attenuation in velocity and small, recirculating flows in the alveolar-like cavities (reproduced with permission from Tenenbaum-Katan et al., 2018).

The nose plays a vital role as the first exposure site between the respiratory system and the external environment during normal breathing (Figure 2A). The nasal passages’ complex, sinuous geometry consisting of multiple pathways (i.e., turbinates) and side chambers (i.e., sinuses) are optimized for the principal tasks of filtering, heating, and humidifying inhaled air destined for downstream transport to the lungs. Concurrently, during such filtering process, the nasal passages can act as a prime infection and replication site for viral airborne pathogens that are trapped and deposit. Here, we developed the *in vitro* nasal compartment chip based on the previously established Carlton-Civic Standardized Nasal Model; an open-source standardized human nose geometry (Liu et al., 2009). Over the years, the model has been established as a geometric standard for both *in vitro* and *in silico* investigations to overcome the challenge of limited comparability between patient-specific nasal anatomies (Brüning et al., 2020). We select a small enough yet relevant section of the Carlton-Civic model (Figure 2B) that can fit on a glass slide but also captures key elements of the nasal anatomy, and specifically the turbinate structures that give rise to unique airflow patterns, including recirculation zones and winding streamlines (Schreck et al., 1993), as illustrated schematically in Figure 2A. Our *in vitro* efforts are driven to integrate the most dominant anatomical structures and airflow features, thus directly influencing the initial site of viral infection and subsequent proliferation pathways via the nasal compartment. In turn, the airflow rate fed into the resulting polydimethylsiloxane (PDMS)-made nasal compartment (Figure 2C) is selected following physiologically relevant references for a typical adult breathing at rest (see details on flow characteristics below and Figure 3).

**TABLE 1:** Characteristic properties and flow parameters of the multi-compartment *airway-on-chip* platform.

Compartment	Units	Nasal	Bronchial	Acinar
Inner volume	V [mm^3^]	506.07	138.26	3.24
Total inner surface area	S [mm^2^]	168.69	75.97	32.4
Height	L [mm]	3	1.82	0.1
Flow rate	Q [ml/min]	6.5	6.5	0.2
Inlet diameter	D [mm]	1	1	1
Inlet Reynolds number	Re_*inlet*_	10	10	0.3
Lung generation	G	-	12–14	20–23

#### Bronchial Compartment Design

The bronchial *airway-on-chip* compartment is based on a recent design from our own group and previously introduced for *in vitro* cytotoxicity assays following realistic *in situ*-like inhalation exposure to immunogenic airborne particulate matter (Elias-Kirma et al., 2020). Briefly, the bronchial geometry consists of a planar, symmetric airway tree spanning three bifurcating generations with a total of four distal branches (Figure 3B). The underlying generic airway tree geometry is based on well-established anatomical models of Weibel (1965) and Horsfield et al. (1971). Representative of small bronchial airway branches of the conducting region of the lungs, the model’s primary airway diameter is 2.5 mm, along with an idealized constant planar bifurcating angle of 60° across all generations. For further details on the airway tree geometry (see Elias-Kirma et al., 2020).

#### Acinar Compartment Design

The acinar airway compartment is based on a recent microfluidic *airway-on-chip* developed in our group (Tenenbaum-Katan et al., 2018) and used to explore inflammatory endpoints following the inhalation of lipopolysaccharide (LPS)-laden nebulized aerosols to simulate bacterial infection *in vitro* (Artzy-Schnirman et al., 2019b). The general acinar tree design features a multi-generation asymmetrically bifurcating network with alveolated airways that span several generations. Briefly, the device holds one inlet airway splitting into six branching generations of 170 μm width and 100 μm height channels, with spherical-like cavities mimicking alveoli of 155 μm diameter; the dimensions of the acinar airway chip are selected to match realistic anatomical dimensions pertinent to the distal respiratory regions of the lungs (Artzy-Schnirman et al., 2019b; Sznitman, 2021). Note that the microfluidic chip provides equal airflow to each terminal end of the model by adjusting the length of a channel connecting the unified outlet of the device and the last generation of the acinar tree (Figure 3C); this design guarantees equal airflow at each terminal end of the acinar model (for further details, see Artzy-Schnirman et al., 2019b).

### Device Fabrication

The nasal and bronchial airway compartments were made using 3D-printed molds filled with polydimethylsiloxane (PDMS) that were subsequently broken apart and removed once the PDMS was entirely cured. Molds were first created using computer-aided design (CAD) (Solidworks 2020, Dassault Systems) before exporting in raw, unstructured triangulated surface format (STL); a format suitable for 3D printing and provided as open-source files in Supplementary Material. STL mold files were prepared (Preform, Formlabs) and subsequently 3D printed in-house via stereo-lithography (Form 2, Formlabs) and made available in the SM. Manufacturer post-print processing instructions were followed, i.e., rinsing in isopropyl alcohol (IPA) and removal of supports, except no post-curing was performed to avoid further hardening the mold material. Liquid PDMS (Dow Corning, Sylgard184) was mixed with a curing agent per the manufacturer’s instructions (1:10 mass ratio) and poured into the molds. Curing was done in ambient (i.e., room temperature) conditions overnight. Subsequently, the 3D-printed mold material was removed leaving a transparent PDMS phantom compartment. Note that the airway inlet and outlet in the nasal compartment are already part of the mold, whereas in the bronchial chip compartment the inlet and outlet are created using a biopsy punch of 1 mm size (Miltex, 3331; see Figure 1B).

Concurrently, the acinar airway tree compartment was fabricated using standard soft-lithography techniques combined with a modified method for master production using dry reactive ion etching (DRIE) of silicon on an insulator wafer to manufacture the small (< 100 μm) features characteristic of the acinar model, as previously described (Artzy-Schnirman et al., 2019b). Briefly, the resulting models were used as a master template for PDMS casting. PDMS mixed with the curing agent was poured on the template, and baked for 1 h at ∼65°C (or overnight at RT). Cured PDMS was subsequently peeled from the mold and punched using a biopsy punch of 1 mm size to create inlet and outlets (Figure 1B).

To assemble completely each individual compartment, a 10 μm thick polyethylene terephthalate (PET) membrane with 0.4 μm pore size (Corning, CLS3450) was bonded to the PDMS channel compartment (i.e., apical compartment) using a “stamping” technique such that channels were irreversibly sealed. Thereafter, the structure was placed using the stamping method on a PDMS well to be filled with culture media (i.e., basal compartment). Finally, a microscope glass slide (Paul Marienfeld, 1000412) was cleaned with ethanol and bonded to the device’s bottom side (reservoir), as shown in Figure 1C. The structure was cured at 65°C for 1 h to complete bonding. We note that only the model’s apical side was sealed to the PET membrane (without the PDMS-glass reservoir) when used for permeability assays (see details below).

### Flow Setup and Characterization

To establish the multi-compartment model’s relevance for recapitulating physiological airflows in the apical compartments of the three selected regions of the lungs, we conducted airflow simulations using computational fluid dynamics (CFD), as shown in Figures 3D–F. This approach gives prior qualitative and quantitative insight into the airflow characteristics anticipated across the individual airway compartments. Briefly, each of the apical airway geometries was meshed with tetrahedral cells using a commercial meshing software (ANSYS, ICEM v18) from the CAD geometry files used in the device fabrication pipeline. Next, a commercial flow simulation software (ANSYS, Fluent v19.2) was used to solve the governing physical equations (i.e., mass continuity and momentum conservation) resolving the airflow fields inside the airway volumes. Here, we simulated steady-state airflow conditions (i.e., equivalent to a steady inhalation) by imposing predefined flow rates (see Table 1) at each compartment inlet and corresponding pressure conditions at the outlets. These simplified conditions were chosen to mimic average respiratory flow rates that may be anticipated within these compartments based on either their respective volume (e.g., the nasal chip’s volume relative to the entire nasal cavity) or anatomical location along the respiratory tract (i.e., lung generation number for the bronchial and acinar chips). While more complex airflows can be introduced to more closely mimic *in vivo* inhalation (e.g., see previous work modeling cyclic ventilation at varying frequencies and flowrates; Nof et al., 2020), we chose here a simplified approach analogous to the perfusion setup employed in our exposure assays that features a nebulizer providing a constant flow output (see details below).

We note here that our *in silico* results are limited to simulations of the inhalation airflows anticipated in each of the compartments and do not encompass Lagrangian particle tracking or ensuing aerosol deposition characteristics. We have previously explored, both in simulations and experiments, characteristics of aerosol deposition of airborne particles in similar bronchial (Elias-Kirma et al., 2020) and alveolar (Fishler et al., 2015) airway models that address the mechanistic determinants of aerosol deposition in *airway-on-chips*. While particle shape, size and density are known to significantly affect deposition patterns within the respiratory tract (Shachar-Berman et al., 2019, Shachar-berman et al., 2020; Koullapis et al., 2021), an extensive study of particle dynamics within our multi-compartment model lies beyond the scope of this work.

To visualize airflow patterns and deliver intuition for possible pathways of particulate matter within the respiratory flow, interpolated streamlines are plotted in Figures 3D–F and colored according to the magnitude of the velocity field. Briefly, in the nasal chamber a recirculation zone is apparent and located in a pocket formed by the turbinate structure (Figure 3D), thereby extending airborne travel time due to reduced flow velocities and longer travel paths inside the cavity before exiting via the outlet port. These flow characteristics recapitulate some of the complexity of nasal flows that may play a role in viral transmission pathways, considering the nose is often regarded as a hospitable replication site. However, we remark that under natural breathing conditions *in vivo*, air does not directly transit through tubes connecting the nose and other regions of the human airways. In contrast, this represents an engineering necessity and limitation featured within our platform that introduce flow artifacts requiring careful further considerations. In the nasal compartment, we see a short jet flow exiting the tube-shaped inlet port resulting in a pair of vortical structures; a well-known fluid dynamics’ characteristic of elongated tube flow emptying into larger ambient spaces. While *in vivo*, the nasal turbinate structures similarly divert flow via impaction, which may lead to vortical or rotational flows, their relative location and sizes likely differ significantly from those introduced here in our platform, which artificially introduces a tube a short distance from a turbinate-like shape within a small section of the nasal cavity.

In the bronchial compartment (Figure 3E), airflow enters through the top of the PDMS compartment before branching twice through two generations of daughter branches and converges again to exit through an outlet passage. Flow characteristics in this airway structure are typical of symmetrically bifurcating airway geometries (see above section on the compartment design), with velocities reduced in more distal airway generations as the flow is split into smaller (in cross-section) but collectively larger (in volume) passages; a direct outcome of mass conservation across a bifurcating tree structure. Note that for small micron-sized aerosols subject to gravitational sedimentation as the leading deposition mechanism, deposition outcomes (not simulated here) have been recently shown to follow a monotonically decreasing trend as velocity decreases with each increasing airway generation number (Elias-Kirma et al., 2020). Flow velocity contours in the acinar compartment (Figure 3F) are similarly characterized by a gradual decrease in velocity magnitudes with each increasing acinar generation. Concurrently, small, recirculating flows in the alveolar-like cavities are present along the alveolated airway channels. Further details on the flow characteristics, including aerosol deposition, in microfluidic acinar airways are available elsewhere using the same geometry (Sznitman, 2021).

Due to high pressure buildup in the small tubing and syringe tip ports (< 1 mm) used in our platform, we could not directly provide high (> 1 ml/s) flow rates into the nasal compartment’s inlet and instead chose a flow rate based on the downstream bronchial compartment that would work well with ordinary laboratory equipment such as a small syringe pump or peristaltic pump (i.e., larger flowrates may for example require ventilators). Thus, we selected a flow rate of 6.5 ml/min (see Table 1) corresponding to a Reynolds number of ∼10 which is typical for the mid-bronchial regions (e.g., 12th generation of the airway tract). We note that the entire Carlton-civic standard nasal model is ∼13.2 ml in volume, while our nasal chip comprises 0.5 ml and represents only 3.5% of the nasal chip volume. Assuming a linear relationship between volume and flow rate (i.e., nasal airflow during rest is laminar and incompressible (Zwicker et al., 2018)), an input flowrate of 6.5 ml/min (i.e., 0.1 ml/s) to the nasal chip would translate to approximately 3 ml/s in a full adult. Unilateral (i.e., from one nostril) nasal airflow rates in adults are reported to vary approximately between a resting rate of 80–200 ml/s and upwards of 1,000 ml/s during physical exertion, with peak Reynolds numbers ranging from several hundreds to thousands (Li et al., 2017). Significant inter-individual nasal anatomical variation results also in diverse air flow rates and due to the cyclic rhythm of breathing (i.e., oscillations between inhalation and exhalation), actual flowrates fluctuate between 0 (at the reversal point between breathing phases) and the maximum reported typical rates. Therefore, while the nasal flows used in our experiments are typically lower than those expected in the nasal passages, they lie nevertheless within the physiological range of resting activity.

Next, flowrates in the bronchial and acinar chips are selected based on typical average physiological flowrates under quiet breathing conditions in an adult human (see Table 1 for selected values at the inlet of each compartment). Airway generation numbers in the bronchial (12–14) and acinar (20–23) chips are given as approximative (Weibel, 1965). Equivalent Reynolds numbers are calculated using the average flowrates, diameter of inlet ports (1 mm), and the rheological properties of ambient air (kinematic viscosity of 1.5 10^−5^ m^2^/s). A 6.5 ml/min (0.1 ml/s) air flowrate through a 1 mm inlet translates to a Reynolds number ∼10, which is appropriate for the 12th generation bronchial airway generation. Note that while the nasal and bronchial apical compartments are connected directly (i.e., flow rate is conserved), air flow continuing from the bronchial to the acinar apical compartment is split by a flow diverter, which releases pressure to the ambient environment thereby reducing the flowrate into the acinar chip (see details below). Following the Y-joint diverter, flow measurements reflect a reduced input flowrate of approximately 0.2 ml/min (i.e., 0.003 ml/s) corresponding to an inlet Reynolds number of ∼0.3 at the first generation of the acinar tree and well in line with characteristic acinar flows (Sznitman, 2013, 2021). This implies that only 3% of the flow continues past the Y-joint to the acinar compartment, due to the pressure gradient between the open end (ambient, low pressure) and high pressure from an increasingly smaller passage in the acinar chip.

### Perfusion System

**FIGURE 4:**
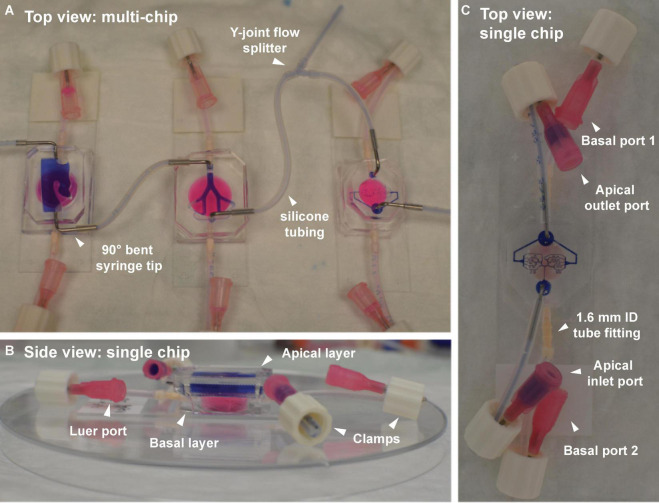
Airflow (apical) and fluidic perfusion (basal) setup for the complete multi-compartment airway-on-chip platform, including silicone tubing and connector fittings. **(A)** Top view of the three airway compartments, with apical partitions connected in series by tubes. A Y-joint tube fitting allows partial bleeding of the airflow between the bronchial and acinar compartments, necessary for reducing the flowrate to the acinar region to lie within a physiologically relevant range (see Table 1). The basal partitions in each chip (filled with pink dye) are fitted with straight nylon tube-to-tube connectors. **(B)** Side view of a single chip (nasal model shown here) highlighting the apical layer (filled with purple dye) atop the basal layer (filled with pinkish dye). Other than when perfusion between the consecutive models is performed, ports remain closed using clamps fitted over a bent section of the tubing near the Luer port end piece. **(C)** Top view of a single representative chip (i.e., acinar compartment shown here) demonstrating the port and tubing configuration used outside of perfusion experiments (e.g., during incubation, microscopy, etc.).

Each of the three airway compartments was connected via silicone tubing (Cole-Parmer, EW- 95702-00), allowing independent perfusion of maintenance media (i.e., culture medium and air) or experimental inputs such as viral-laden aerosols (apical side only; see results below) applied to the nasal compartment and subsequently transmitted to bronchial and acinar models (see Figure 4). During experiments, a Y-joint tube-to-tube connector (Nordson Medical, Y210-1) was used to “bleed off” or reduce the flowrate fed to the acinar model, based on the physiologically relevant range (see Table 1) mimicking airflows at such airway depths and in line with previous microfluidic efforts (Sznitman, 2021).

For the convenience of cell culture maintenance and to restrict the spread of potential infections, each compartment is initially handled separately (see Figure 4B) before conducting any experimental protocol (see below). Briefly, the apical compartment (filled with blue dye for contrast visualization in Figure 4) is accessible via two Luer ports connected via silicone tubing to holes in the PDMS material, made with a 1 mm biopsy punch and strengthened with either straight (nasal compartment only) or 90 deg. bent 18-gauge syringe tips (Techcon, TE720050B90) for robustness and protection from tearing PDMS material. Note that the metal syringe tips are stripped from their original plastic hubs and sterilized before integrating with the model.

Closing all Luer ports to either apical or basal compartments (see Figure 4C), is accomplished via insertion of male Luer plugs (not shown) or clamps. While plugs (Nordson medical, LP4-1) are robust and stable, simply bending the tubes and applying a clamp (Nordson Medical, FSLLR-1), proved quicker and more amenable to the maintenance of sterility necessary for cell culture. Similarly, the basal compartment (filled with pinkish dye in Figure 4) is accessed via two Luer ports connected via silicone tubing to holes in the PDMS material and strengthened with nylon, straight barbed tube-to-tube connectors (Nordson Medical, N210-1); these latter connectors are of larger inner diameter than the apical side’s syringe tips thus allowing for easier perfusion of culture medium (see Figure 4C).

### Cell Culture

To capture key aspects of the cellular makeup relevant to our principal lung regions, we use well-established airway epithelial cell lines (Selo et al., 2021). Our strategy thus provides a human-relevant pulmonary benchmark that can be adapted for and compared with future studies using primary cells and patient-derived samples. We recall that cell lines afford increased control over experimental parameters with reduced genetic variation and facilitated culture requirements (Kaur and Dufour, 2012) to culture, maintain and transduce, and thus may, be used to mimic some of their tissue origin behavior and characteristics (Masters, 2000). With that said, cell lines still hold limitations that should be taken into consideration. Notably, they have been shown to have genetic and phenotypic alterations form their origin tissue (Alge et al., 2006; Pan et al., 2009), compared to primary cells that are isolated directly from the tissue of interest, and thus capture more closely cellular heterogeneity.

As representative of our model compartments, we chose to work with three cell lines originating from matching tissues: (i) RPMI-2650 is an epithelial cell line derived from the human nasal septum, (ii) Calu-3 are isolated from human lung adenocarcinoma and resemble the bronchial epithelium (Kreft et al., 2015a), and finally (iii) the alveolar epithelial hAELVi cell line originated from alveolar type I cells (Kuehn et al., 2016).

**FIGURE 5:**
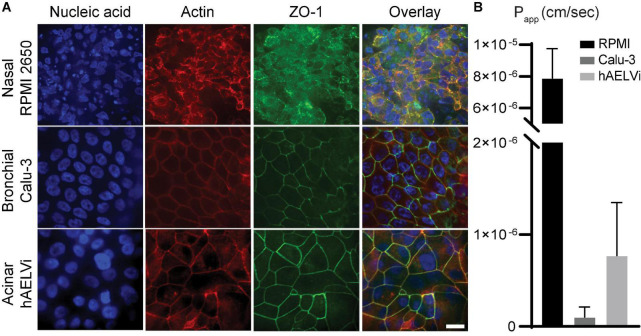
Characterization of human-relevant cell lines mimicking relevant airway epithelial cells of the respective lung compartments. **(A)** Immunofluorescence micrographic views for three cell lines: RPMI 2650 seeded in nasal models, Calu-3 seeded in bronchial models, and hAELVi seeded in acinar models, respectively, and immunolabeled with: 4′,6-diamidino-2-phenylindole (DAPI) for nucleic acid staining (blue), phalloidin for actin filaments staining (red), anti-Zonula occludens-1 (ZO-1) (green), with an overlay view. The scale bar is 20 μm for all images. **(B)** Transport assay of fluorescein sodium salt (FluNa), a functional permeability test for cellularized models, conducted ∼3 weeks post-seeding. RPMI 2650 shows high apparent permeability (Papp) value of 8 × 10^– 6^ cm s^– 1^ indicating poor barrier properties, while Calu-3 and hAELVi yield low Papp values of 1 × 10^– 7^ and 7 × 10^– 7^cm s^– 1^, respectively, indicating effective airway barriers.

To first evaluate cell line characteristics, we made use of established epithelium integrity characterization methods, in addition to staining techniques using 4′,6-diamidino-2-phenylindole (DAPI) as nuclear stain, phalloidin as actin filaments stain, along with tight junction anti-Zonula occludens-1 (ZO-1) antibody followed by fluorescent secondary antibodies. Initially, we conducted a standard evaluation in 24 transwells for both liquid-liquid interface (LLI) and ALI conditions (see Supplementary Figures 1–3) using cell inserts. Next, we repeated such evaluations directly in our PDMS-made *airway-on-chip* models (Figure 5). Comparing staining results in both (ALI) inserts chip models reveals strong qualitatively similar cell behavior across, suggesting compatible growth conditions in the models.

## Materials and Integrity Assays

### Cells

RPMI2650 (ATCC, CCL-30) and Calu3 (ATCC, HTB-55) were cultured in ATCC-formulated Eagle’s Minimum Essential Medium (Biological Industries, 01-040-1A) supplemented with fetal bovine serum (FBS) (Biological Industries, 04-127-1A) to a final concentration of 10%, 1% L-glutamine (Biological Industries, 03-020-1B) and 1% antimycotic antibiotics (Sigma-Aldrich, A5955). The medium was changed every other day. When cells reached 90% confluency, they were trypsinized, using Trypsin EDTA Solution B (0.25%), EDTA (0.05%) (Biological Industries, 03-052-1B), and used for maintenance or experiments, as described below. hAELVi cells (InSCREENeX, INS-CI-1015) were cultured in a small airway epithelial cell growth medium (SAGM) BulletKit (Lonza CC-3118) supplemented with 1% FBS and 1% penicillin/streptomycin (P/S) (Life Technologies, 15140-122). Prior to seeding, flasks were coated with coating buffer; (1% (v/v) fibronectin (Corning, 33016015) and 1% (v/v) collagen (Sigma, C4243) for 2 h in 37°C or overnight in 4 °C. The medium was changed every 2–3 days. When cells reached 90% confluency, they were trypsinized using Trypsin-EDTA (0.05%) (Gibco, 25300054) and used for maintenance or experiments, as described below. Cells were incubated in humidified incubators at 5% CO_2_, 37°C at all times.

For SARS-CoV-2 virus incubation (see assays below), the Vero E6 (ATCC, Vero C1008, CRL-1586) cell line was used. Briefly, cells were grown in Dulbecco’s modified Eagle’s medium (Biological Industries, 01-055-1A) containing 10% Fetal bovine serum (Biological Industries, 04-127-1A), MEM non-essential amino acids (Biological Industries, 01-340-1B), 1% L-glutamine (Biological Industries, 03-020-1B), 1% penicillin/streptomycin (Biological Industries, 03-031-5C).

### Viruses

SARS-CoV-2 viruses (GISAID accession EPI_ISL_406862) were kindly provided by Bundeswehr Institute of Microbiology, Munich, Germany. Virus stocks were propagated (4 passages) and tittered on Vero E6 cells before it was used. Handling and working with SARS-CoV-2 virus were conducted in a BSL3 facility in accordance with the biosafety guidelines of the Israel Institute for Biological Research (IIBR).

Recombinant lentiviral particles were generated using a Lenti-X Packaging Single Shots (VSV-G) System (Takara Bio USA, #631275). This system produces replication-incompetent VSV-G pseudotyped lentiviruses. The lentiviruses were generated according to the manufacturer’s instructions. Briefly, the expression vector for the fluorescent protein tdTomato (Takara Bio USA, #632564) was transfected, in a Lenti-X Packaging Single Shot, into the Lenti-X 293T packaging cells (Takara Bio USA, #632180). After 48 h, the lentiviral supernatant produced by the transfected packaging cells was collected and filtered through a 45 μm filter (Merck, Millipore SLHP033RS) to remove cellular debris, and then used to transduce the devices.

### Insert Cell Culture

RPMI 2650 (5 × 10^5^ cells) and Calu-3 cells (1 × 10^5^ cells) were seeded on uncoated transwells polyester membranes (Corning 3470; growth area 0.33 cm^2^; pore size 0.4 μm), while hAELVi cells (1 × 10^5^ cells) were seeded on precoated transwells (as described previously). All cells were seeded under liquid-liquid interface (LLI) conditions. 200 μL of culture medium was perfused on the apical side and 500 μL through the basolateral side. The medium was changed every second day. For ALI conditions, the medium from the apical compartment was aspirated 2 days post-seeding, and the cells were further fed from the basolateral compartment with 500 μL of the medium.

### Microfluidic Cell Culture

The devices were sterilized following three rounds of exposure to ultraviolet light (254 nm), 15 min each. Afterward, the apical side of acinar devices was incubated with coating buffer for 2 h in 37°C, 5% CO_2_, and 95% humidity, washed with PBS, and seeded without drying the membrane. RPMI 2650 (1 × 10^6^ cells ml^–1^), Calu-3 (1 × 10^6^ cells ml^–1^), and 10 μL of hAELVi cells suspension were seeded on the apical side of the membrane of the nasal, bronchial, and acinar airway chips, respectively. All basolateral compartments were filled with suitable growth medium. After 48 h of seeding, the apical compartment was washed with fresh media to wash out non-attached cells. Following 48 h in LLI conditions, the medium from the apical compartment was aspirated, allowing the cells to grow at ALI conditions. Next, the cells were supplied medium once a week from the basolateral compartment by withdrawing the liquid from the basolateral side and injecting fresh medium.

### Transport Studies

Inserts and devices were seeded with cell cultures as described above. The cells were allowed to grow for about 3 weeks (in ALI when confluency is reached). Transport experiments were then performed as previously described (Elbert et al., 1999). Briefly, the cells were washed twice with prewarmed Krebs–Ringer Buffer (KRB: NaCl 142.03 × 10^–3^ m, KCl 2.95 × 10^–3^ m, K_2_HPO_4_*3H_2_O 1.49 × 10^–3^ m, HEPES 10.07 × 10^–3^ m, d-glucose 4.00 × 10^–3^ m, MgCl_2_*6H_2_O 1.18 × 10^–3^ m, CaCl_2_*2H_2_O 4.22 × 10^–3^ m; pH 7.4), and incubated in KRB for 45 min. Next, the medium was aspirated. Fluorescein sodium salt (FluNa) (Sigma-Aldrich, F6377) was added to the apical compartment (first diluted in KRB buffer for a final concentration of 0.01 μg ml^–1^), and KRB was added to the basolateral compartment. The devices were next placed in the incubator, and 30 μL samples were taken every 30 min from the basolateral compartment only and transferred into a 384-well plate to measure FluNa concentrations. Sampled volumes were refilled with 30 μL KRB. The samples in the 384-well plates were measured with a Synergy H1 microplate reader (Agilent BioTek, #1902191C) using wavelengths of 488 nm (excitation) and 530 nm (emission).

### Transepithelial Electrical Resistance Measurement

Transepithelial Electrical Resistance (TEER) was measured as previously described (Kuehn et al., 2016). Briefly, 24 h post cells seeding on transwells, and every other day, per the routine care, media was aspirated, the apical side was refilled with 200 μL prewarmed medium, and the basolateral compartments were filled up to a final volume of 500 μL. Following 1 h of incubation, TEER was measured in all samples using a dedicated epithelial volt-ohm meter (Millicell ERS-2) equipped with chopstick electrodes (Millicell, MERSSTX01). In transwells grown under ALI conditions, media of apical compartment was aspirated immediately following the measurements. The electrical resistance was calculated by subtracting the value of blank inserts containing medium from all samples and multiplication with the cultivation area of the inserts (i.e., 0.33 cm^2^).

## Biochemical Assays

### Measurement of Viral RNA

A total of 200 mL of samples from each compartment were added to LBF lysis buffer (supplied with the kit) and viral RNA was extracted using RNAdvance Viral Kit (Beckman Coulter) and further processed on the Biomek i7 Automated Workstation (Beckman Coulter), according to the manufacturer’s protocol. Each sample was eluted in 50 μL of RNase-free water. Realtime RT-PCR assays, targeting the SARS-CoV-2 E gene, were performed using the SensiFAST Probe Lo-ROX One-Step kit (Bioline). The final concentration of primers was 600 nM and the probe concentration was 300 nM. Primers and probe for the E gene assay were taken from the Berlin protocol published in the World Health Organization (WHO) recommendation for the detection of SARS-CoV-2. Thermal cycling was performed at 480°C for 20 min for reverse transcription, followed by 95°C for 2 min, and then 45 cycles of 94°C for 15 s, 60°C for 35 s. Cycle Threshold (Ct) values were converted to calculated plaque-forming unit (PFUs) with the aid of a calibration curve tested in parallel.

### Plaque Forming Unit Assay

Vero E6 cells were seeded in 12-well plates (5 × 10^5^ cells/well) and grown overnight. Dilutions of SARS-CoV-2 were prepared in MEM containing 2% FCS with NEAA, glutamine, and P/S, and used to infect Vero E6 monolayers (500 μL/well). Plates were incubated for 1 h at 37°C to allow viral adsorption. Then, 1 ml/well of overlay [MEM containing 2% FBS and 0.4% Tragacanth (Merck)] was added to each well and plates were incubated at 37°C, 5% CO_2_ for 48 h. The media was then aspirated, and the cells were fixed and stained with 500 μL/well of crystal violet solution (Biological Industries). The number of plaques in each well was determined.

### Cytokine Secretion Assay

Cell culture supernatants were assayed using ELISA for Interleukin-6 (IL-6) (Thermo Fisher Scientific, #88-7066-88) following the manufacturer’s instructions. Briefly, the basal media of exposed inserts and models were collected, centrifuged to discard precipitated cell fractions, and diluted if necessary. Samples were added to pre-coated plates-conjugated to capture antibodies and blocked to exclude non-specific interactions. A detection antibody was added to generate sandwich reaction. Avidin-HRP was added, followed by TMB solution to generate detection reaction, which was stopped using sulfuric acid (2N H_2_SO_4_) upon saturation of calibration wells.

### Immunofluorescence Microscopy

Directly after cell fixation using 4% PFA, cells were treated with 0.05% Triton X-100 (Sigma–Aldrich, T8787) for 3 min at room temperature (RT) to increase membrane permeability. Afterward, cells were blocked for non-specific binding using 2% BSA (MP Biomedicals, 02160069-CF) for 1 h at RT. For F-actin staining, cells were incubated with Alexa Fluor 568 Phalloidin (Thermo Fisher Scientific, A12380) diluted with PBS (ratio of 1:200) for 40 min at RT. For DAPI nucleic acid staining, cells were incubated with DAPI solution (ThermoFisher Scientific, D1306), diluted with PBS (ratio of 1:500) for 5 min at RT. For tight junction protein-1 (ZO1) staining, cells were incubated with the primary antibody rabbit anti-ZO1 (Thermo Fisher Scientific, 617300) diluted with BSA (ratio of 1:200) overnight at 4°C, followed by incubation with secondary antibody Alexa Fluor 488 anti-rabbit (Jackson ImmunoResearch, 111-545-144) diluted with BSA (ratio of 1:500) for 1 h at RT. After each step, cells were washed three times with PBS. Finally, fluorescent immunostaining confocal microscopy imaging was performed (Nikon Eclipse Ti with spinning disk).

## Results

### Epithelium Integrity Characterization

We conducted two well-established integrity assays (Sakagami, 2020; Artzy-Schnirman et al., 2021) in 24 transwells to evaluate epithelial barrier functionality. First, we measured trans-epithelial electrical resistance (TEER) for a span of 3–4 weeks during cell culture. This was followed by tracking fluorescein sodium salt transport through the apical to basal chamber, and subsequently extracting the apparent permeability coefficient (Papp) across the airway epithelium. RPMI 2650 results revealed a culture of multi-layer clusters, with ambiguous ZO-1 formation (Supplementary Figure 1), low TEER and high Papp values (Supplementary Figures 4A,D), in accordance with other *in vitro* characterization assays (De Fraissinette et al., 1995; Psimadas et al., 2012). Conversely, Calu-3 and hAELVi cells exhibited a uniform polarized monolayer, with clear tight junction staining (Supplementary Figures 2, 3), corresponding to high TEER (Supplementary Figures 4B,C) and very low Papp values (Supplementary Figure 4D), underlining a functional barrier and in line with previous works (Kreft et al., 2015a; Kuehn et al., 2016). Furthermore, for all cell lines, we note that cultures grown at the ALI showed comparatively higher TEER and Papp values, in addition to visually clearer and smoother staining resulting from a more polarized epithelial cell organization, in contract with more noisy and blurry results for cultures maintained in LLI; a well-known feature of epithelial airway cells grown under more physiologically faithful conditions (Sakagami, 2020; Artzy-Schnirman et al., 2021).

Similarly, we examined cell behavior in our microfluidic models, grown at ALI conditions for a period of 3 weeks. We stained for DAPI, phalloidin, and ZO-1, in addition to FluNa transport assays. The devices used for barrier functionality evaluation consisted of the model’s apical compartment sealed to the PET membrane, placed in 24 mm well (of a 6 well plate) in lieu of the basal compartment and medium reservoir, rather than PDMS-glass reservoir. As previously described (Figure 1), the upper side of the apical compartment is sealed with PDMS (except for the openings in place at the inlet and outlet holes); this setup is inconvenient for conducting routine TEER measurements. However, in the present modified form, the basal reservoir is readily accessible for media sampling necessary for transport assays. Hence, we examined the models’ barrier functionality with the permeability assay only, shown to correlate with TEER behavior in transwell inserts. Similar to our previous observations, RPMI 2650 in nasal models formed cell clusters, blurry images, incomplete ZO1 staining and high Papp values. This comes in contrast to confluent monolayer formation, unified tight junctions, as well as low Papp values reported in both bronchial Calu-3 models and acinar hAELVi models (Artzy-Schnirman et al., 2019b; Figure 5B). We also compared barrier characterization for chip models in LLI and observed similar patterns to those seen in transwells (Supplementary Figure 4E).

### Development of the Multi Compartment Platform

The methods described herein establish our multi-compartment *airway-on-chip* platform with endpoints aimed at recapitulating three representative elements of the respiratory system underlining both physiological (i.e., airflow patterns) and biological (i.e., cellular) relevance. In what follows, we now exemplify the usability of the platform for some selected, but not exhaustive, *in vitro* endpoints linked to viral airborne transmission of respiratory infections. In the first proof-of-concept embodiment, we measured the transmission of the SARS-CoV-2 through the platform, demonstrating the physical capabilities of the platform for recapitulating *in vitro* an attenuated viral transmission pathway through the respiratory tract following infection in the nasal airways. The second assay employed transduced non-replicating Lenti-X-Lentivirus to isolate and measure the extent of airflow-mediated viral deposition stemming from an initial infection site in the nasal compartment. Finally, in the third proof-of-concept, we measured the compartment-specific inflammatory cellular response to inhaled viral transmission in the airways using a non-replicating viral simulant (i.e., Poly I:C) and biochemical analysis of cytokine secretion.

**FIGURE 6:**
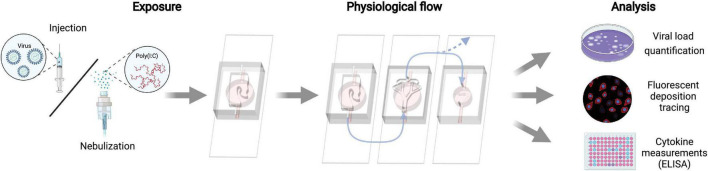
Schematic of the experimental proof-of-concept protocols for inhalation assays. Exposure is limited first to the nasal compartment, either via direct instillation of the virus or nebulized Poly I:C viral simulant. Following exposure, all three apical compartments are connected in series to airflow supplied via a peristaltic pump. Note that airflow to the acinar compartment is reduced to a physiologically relevant range in the deep lungs (see Table 1). Each compartment is analyzed separately for viral quantity, deposition sites, and cellular cytokine secretion to the basal media.

We underscore that in all three proof-of-concept assays, we attempted to follow a realistic pulmonary infection pathway, initiating in the nasal compartment as the acknowledged initial exposure site. Subsequently, viral (or viral-like) laden aerosols were sequentially transmitted to the bronchial and acinar compartments subject to air flow-mediated mechanisms (Figure 6).

### Syndrome-Related Coronavirus 2 Deposition in the Multi-Compartment *Airways-on-Chip* Platform

For this proof-of-concept assay, we used a human respiratory virus (SARS-CoV-2) where airflow transmission was conducted using a peristaltic pump (MRC, PP-X-575). The apical compartment of the nasal device was first injected with SARS-CoV-2 (1.2 × 10^6^ PFU). Afterward, the apical compartments of all devices were sequentially connected. The Peristaltic Pump was connected to the apical compartment of the nasal device, and airflow (6.5 ml/min) was applied for 10 min, a relatively short exposure period chosen for safety precaution reasons. Right afterward, apical compartments were washed with MEM containing 2% FCS with NEAA, glutamine, and P/S, and samples were analyzed for viral RNA and PFU.

**FIGURE 7:**
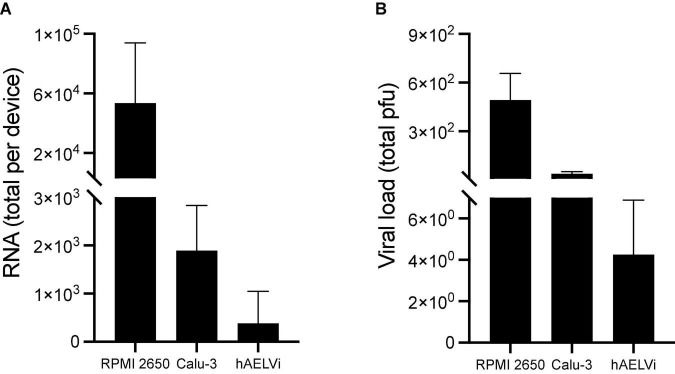
Functional viral transmission of SARS-CoV-2 in the multi-compartment *airways-on-chip*. Compartmental viral load was quantified via **(A)** real-time PCR and **(B)** Plaque-forming units (PFU). In both assays, viral traces were detected in all three compartments, indicating the crosstalk in the chips. A monotonically decreasing viral presence is measured across the model, with considerable reduction observed between nasal and bronchial compartments owing to high deposition in the nasal compartment and tube connections. Further reduction in viral presence is measured in the acinar compartment, likely resulting from aerosol deposition in conjunction with flow diversion resulting from the Y-joint. Data are the average of three experiments presented as mean and the standard deviation (SD).

Both viral RNA and PFU results (Figure 7) support the plausible transmission of viral-laden aerosols from the nasal to bronchial compartments, and subsequently to the acinar unit. We note a stark reduction in viral load that decreases monotonically across the compartmental pathways, where most of the initial load reaches the first exposure site in the nasal passages. The most drastic reduction in viral presence seen in the acinar compartment likely results from aerosol deposition and flow diversion arising at the Y-joint (Figure 6). While the described *in vitro* assay supports and demonstrates the multi-compartmental platform’s utility for delivering viable and functional SARS-CoV-2 airborne particles to the different compartments from a methodological and experimental standpoint, we note that further investigations would be nevertheless required to deliver a broader cellular characterization to test the susceptibility of the cells to the specific virus and the *in vitro* pathology of SARS-CoV-2, or any other virus.

### Transduced Lenti-X-Lentivirus for Non-replicating Inhaled Viral Transmission Simulation

**FIGURE 8:**
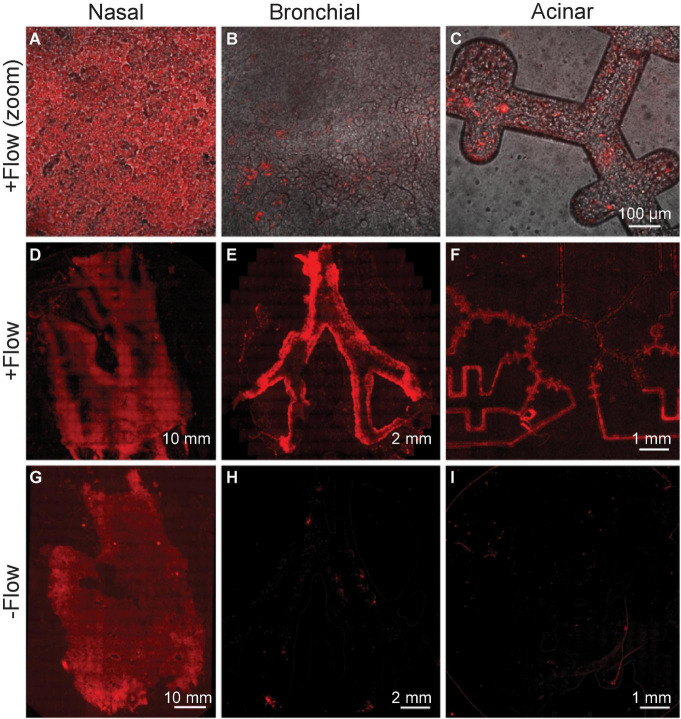
Viral deposition tracing using Lenti-X-Lentivirus, a transduced non-replicating virus. Epithelial cells in each of three compartments are infected with virus expressing red fluorescent protein following exposure to physiological flow conditions. **(A–C)** Top row images (all the same scale) show cell-level magnification, while the bottom rows **(D–I)** show stitched microscopy images to view each compartment entirely. **(D–F)** Infection supports the airflow crosstalk in the chip’s multi-compartments, as the transfer of virus is witnessed in each compartment downstream from the nasal passages. The bottom row **(G–I)** shows the control experiment, in the absence of any airflow applied, and showing thus the lack of airflow-mediated airborne transport. The figure represents one of three experiments yielding similar results.

In this next proof-of-concept assay, we used a transduced non-replicating Lenti-X-Lentivirus to simulate initial viral exposure and transport, isolating the pathways of invading particles in our platform from the effects of replicative viral transmission. We then measured deposition in the three compartments under both physiological airflow and control (i.e., zero flow) conditions, revealing the determining role of airflow-mediated viral deposition in our platform (see Figure 8).

The exposure assay is conducted as follows: 150 μL of lentiviral supernatant was injected into the nasal compartment and set for 15 min (see Figure 6). Meanwhile, bronchial and acinar models were connected, and the whole *airways-on-chip* platform was assembled. Airflow (6.5 ml/min) was applied using a peristaltic pump (Ismatec, ISM597D) to the assembled devices, flowing from the nasal model to the bronchial model and lastly through the acinar model for 72 h in a humidified incubator. The experiment was terminated only 2 h post flow arrest to allow the deposition under gravitational sedimentation of any remaining airborne viral aerosols. In parallel, as a control the nasal model was similarly exposed to the transduced virus and connected in series with other compartments (Figure 6), but no airflow was applied. The devices were imaged using a tile scan with the Axio Observer 7 microscope (Zeiss). Images were processed using ZEN Blue v2.3 (Zeiss).

Our imaging results show that epithelial cells expressing fluorescent protein tdTomato were detected in all three distinctive compartments (Figure 8), supporting the airflow-mediated hypothesis toward transmission of primary viral load to all regions. Concurrently, no fluorescence was detected in either bronchial or acinar models when no airflow was applied, further underscoring the central role of airflow-mediated transmission.

### Poly I:C for Non-replicating Viral Transmission Simulation

In a final proof-of-concept assay, we used Poly I:C, a synthetic double-stranded ribonucleic acid (dsRNA) molecule, to examine the initial cellular inflammatory response upon exposure to virus components as a pathogen-associated molecular pattern (PAMP). Poly I:C molecules are recognized by toll-like receptor-3 (TLR-3) expressed on the surface of epithelial cells, and elicit intracellular signaling pathways, subsequently secreting inflammatory cytokines such as interleukin-6 (IL-6) (Alexopoulou et al., 2001; Akira et al., 2006).

**FIGURE 9:**
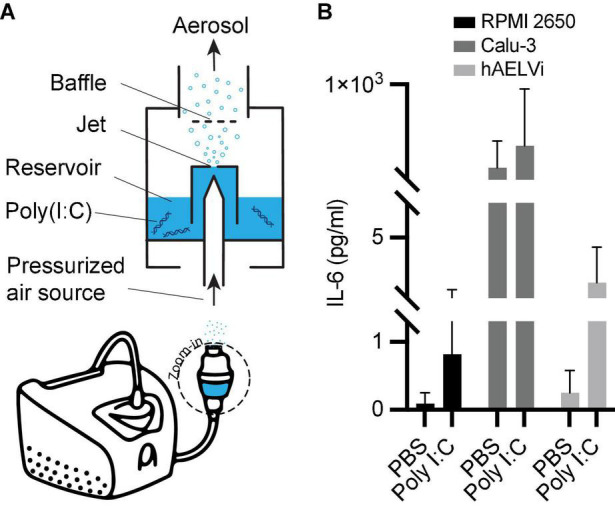
Non-replicating viral transmission simulation using poly I:C and subsequent IL-6 secretions across the three compartments. **(A)** Schematic of the nebulization setup used to supply aerosolized poly I:C to the multi-compartment model. Following exposure, cells are freeze-burst and their media collected for **(B)** differentiated quantification of inflammatory cytokines in each compartment. Interleukin 6 (IL-6) was measured via enzyme-linked immunosorbent assay (ELISA). In all models, an elevation of IL-6 levels can be seen upon exposure to poly I:C compared to PBS exposure as a control. Bars represent the average and error bars of the standard deviation (*SD*) (*N* = 3 for PBS, *N* = 5 for poly I:C).

Using a standard compressor nebulizer (Bi-rich medical, BR-CN116), we supplied aerosolized poly I:C to the nasal compartment. The nebulizer cup (see the enlarged region in Figure 9A) was filled with diluted (100 μg ml^–1^ in PBS) Poly I:C solution and connected via silicone tubing and a 19-gauge syringe tip to the nasal compartment’s inlet port. Following 4 min of aerosol exposure, the nebulizer was turned off, and the platform was left to stabilize for 15 min, during which the bronchial and acinar compartments were connected in series (as shown in Figure 6). Once the multi-compartment platform was fully assembled, physiological airflow was perfused through the model for 72 h using a peristaltic pump (Ismatec, ISM597D). Thereafter, we placed the models in a –80°C freezer to induce freeze-burst of cells and discharge intracellular content. Subsequently, we collected the basal media and quantified the inflammatory cytokine interleukin 6 (IL-6) via Enzyme-Linked Immunosorbent Assay (ELISA). Quantitative analysis (Figure 9B) showed poly I:C induced elevation in IL-6 levels in all cell lines.

Calibration experiments (Supplementary Figure 5) show significant increases in cytokine secretion following direct poly I:C exposure across all cell lines, indicating the biological compatibility of the platform and selected cell populations for measuring inflammatory response to poly I:C. Exposure to aerosols generated via nebulization from equivalent concentrations of poly I:C follow similar patterns of elevated cytokine secretions. However, repeated experiments in RPMI2650 and hAELVi cells generated larger statistical variance indicating a noisier signal. The greater variability of aerosolized poly I:C molecules coming into contact with the cultured epithelial cell populations emphasizes the importance of flow-mediated exposure phenomena for the transmission pathway and the delicate caution needed for interpreting aerosol-based *in vitro* assays.

## Discussion and Outlook

With the Methods presented herein, our multi-compartment *airways-on-chip* is a versatile *in vitro* platform that can be further modulated and adapted for the needs of a wide variety of preclinical research endpoints, including for example questions pertaining to respiratory viral transmission in the human lungs. Specifically, we have provided all the necessary source files, including 3D printing formats needed to fabricate our platform (see Supplementary Data) and perform subsequent calibrations. Our three experimental proof-of-concept embodiments, spanning SARS-CoV-2, a transduced Lentivirus, and dsRNA Poly I:C were selected to showcase several biological and cellular capabilities of our platform as an attractive proof of concept. These include recapitulating physiological and biological characteristics of distinct respiratory tract key regions, alongside the capability to assess viral laden and cellular response in the different respiratory compartments and the opportunity to mimic *in vitro* aspects of airborne crosstalk and transmission in the lungs.

Much of our initial motivation has been rooted in a platform for investigating transmission pathways, cell infection, and inflammation initiation by airborne viruses such as SARS-Cov-2. The compartmentalized, regional representations in each of the three compartments can be leveraged and adapted further for various *in vitro* assays to quantify regional differences in viral exposure under the influences of varying exposure conditions, aerosol dispersion characteristics and related airflow patterns and importantly, distinct epithelial cell populations.

As a first step, we examined the platform’s compatibility for harboring and transporting SARS-CoV-2, of heightened interest amidst the ongoing global pandemic. Downstream transmission of viral particles to distal compartments from an initially isolated nasal compartment supports the model’s crosstalk for airflow-mediated viral transmission, alongside a gradual reduction of viral load. However, the use of SARS-CoV-2 in experiments introduces tremendous safety risks and logistical burden (e.g., PPE, bio-safety rooms) that are intrinsically cumbersome when developing a new *in vitro* platform intended for more general virological studies. Furthermore, we hoped to demonstrate our platform’s utility in laboratory settings that may lack the special safety conditions necessary for handling such dangerous and infections viruses. Consequently, in subsequent experiments, we employed other, less harmful and non-replicating viral proxies such as transduced lentivirus and the viral simulant poly I:C, demonstrating our platform’s broader utility and opening the way for experimental protocols such as aerosolization, that would be limited by safety considerations when handling for example SARS-CoV-2.

We exposed our model to transduced Lenti-X-Lentivirus to estimate the initial dispersion and deposition of viral-laden aerosols, identifying infected cells via fluorescence microscopy. Fluorescent cells were imaged in all compartments, further confirming the crosstalk between the compartments and the existence of a viable transmission pathway. This experiment tracked the viability of the cell monolayer covering the whole model surface throughout and following the exposure event, necessary for subsequent biochemical analyses of cellular response. We note here that our assay demonstrates transmission, deposition, and initial viral or viral-like exposure, while a study of infection as well as the full biologic mode of action lies beyond the scope of this methods paper.

Due to the safety limitations mentioned above, SARS-CoV-2 and lentivirus were injected via a syringe to the nasal compartment in both experiments, in contrast to *in situ* viruses introduced into the respiratory system as aerosols, affecting the transmission mechanism and deposition outcomes (Artzy-Schnirman et al., 2019a). Acknowledging the importance of aerosol effects, and in order to mimic transmission pathways more realistically (Artzy-Schnirman et al., 2019a,b; Wang et al., 2021), we used a nebulizer to create an aerosolized exposure of poly I:C, a synthetic double-stranded ribonucleic acid (dsRNA) molecule that can be handled in a biosafety room 2 in a safety hood. Beyond its safer suitability for nebulization, poly I:C can still be recognized by toll-like receptor-3 (TLR-3) in epithelial cells, thus eliciting inflammatory response and secretion of inflammatory cytokines. Quantifying such cytokines in each compartment may enable to assess the local cellular stress, dependent on each compartment’s particular physiology, airflow pattern, and cellular composure.

Whereas our platform was designed to have a relatively small height to improve optical clarity for microscopy, higher three-dimensionality may be incorporated where imaging is less of a priority or in tandem with more advanced three-dimensional imaging technology. Additionally, more complex flow patterns may be used for perfusing air in the apical compartments to more realistically mimic human breathing, such as using a programmable linear actuator or mechanical ventilator as done in previous works (Nof et al., 2020, 2021).

In parallel, we note that human cell lines differ from cells *in vivo*, both genotypically and phenotypically (Pan et al., 2009) and thus exhibit different functionality. Amongst other, such differences are embodied in the absence of cilia structure and mucus production in both the nasal RPMI2650 (Christiane Schmidt et al., 1998; Kreft et al., 2015b) and bronchial Calu-3 (Foster et al., 2000; Mathias et al., 2002; Fiegel et al., 2003) cell lines, thereby leading to underestimating mucociliary clearance on the distribution of the viral and viral-like particles in the three compartments. In parallel, hAELVi cells are alveolar type I like epithelial cells, and thus cannot produce surfactant (Ferguson and Schlesinger, 2000); a unique composite of lipids and proteins that play a crucial role in initial immunity (Ferguson and Schlesinger, 2000; Glasser and Mallampalli, 2011), that may further affect the ability of infectious particles to attain the cells. Alternatively, primary patient-derived cells can be used when patient variation response is of interest or to improve the biological relevance of the cellular response assays at the cost of throughput. Here, our choice for selecting cell lines lies in their reliability, robustness, and non-patient-specific variability in serving as a benchmark for end users (Hittinger et al., 2015; Sakagami, 2020; Selo et al., 2021), in particular during preclinical *in vitro* stages. Note nevertheless that cellular assays can be adapted to include additional co-cultures, including but not limited to immune cells in the apical compartment (Ainslie et al., 2019; Artzy-Schnirman et al., 2019b) (e.g., macrophages) (Ainslie et al., 2019), but also reconstructing an air-blood barrier (ABB) with endothelial cells cultures on the basal side of the membrane (Barnes et al., 2015a; Artzy-Schnirman et al., 2021).

Lastly, we raise the point that our *in vitro* platform exhibits distinct cellularized airway compartments, connected via silicon tubing exclusively during experimental procedures (see section “Materials and Methods” above). The isolation of the individual compartments during the preparation phase allows for experimental easiness in maintenance as well as during cellular differentiation, and finally during independent compartmental viral load measurements. Furthermore, our platform’s modular “plug and play” design leverages easier adoption and adaptability for other preclinical research needs, specifically with the end user in mind (Ainslie et al., 2019; Artzy-Schnirman et al., 2019a). With such functionalities enabled during experimentation, we note, however, here that these advantages come at the cost of introducing various artifacts relative to the natural *in vivo* lung environment, including foremost a discontinuity in the epithelial lining that is anticipated to affect viral propagation characteristics along a continuous airway tract (Leung, 2021; Wang et al., 2021). Notably, this includes innate clearing and defense mechanisms (Nicod, 2005) via mucociliary clearance in the conducting regions of the lungs, as well displacements of the surfactant-rich liquid lining layer in the deep respiratory regions. With these limitations in mind, our *in vitro* efforts offer to the best of our knowledge the first *airway-on-chip* attempt to deliver a more comprehensive recapitulation of the broader lung regions via key lung compartments that breaks away from current state-of-the-art individual isolated *lung-on-chip* models (Benam et al., 2015; Artzy-Schnirman et al., 2021).

Altogether, our efforts deliver an open-source *in vitro* multi-compartment platform available to end users across the academic and biopharmaceutical communities that can be utilized and adapted further as a powerful tool in preclinical research for investigating amongst other respiratory infections, host-pathogen interactions as well as potential drug screens and discovery endpoints.

## Data Availability Statement

The original contributions presented in the study are included in the article/Supplementary Material, further inquiries can be directed to the corresponding author.

## Author Contributions

EN, HZ, and AA-S: methodology. EN and SB: software. EN and AA-S: validation. EN, HZ, AA-S, OM, MB, DG, and AB-D: investigation. SE-K: resources. EN and HZ: writing–original draft. EN, HZ, AA-S, and JS: writing—review and editing. AA-S, SL, NK, AO, and JS: supervision. AA-S, AO, and JS: funding acquisition and conceptualization. All authors contributed to the article and approved the submitted version.

## Conflict of Interest

The authors declare that the research was conducted in the absence of any commercial or financial relationships that could be construed as a potential conflict of interest.

## Publisher’s Note

All claims expressed in this article are solely those of the authors and do not necessarily represent those of their affiliated organizations, or those of the publisher, the editors and the reviewers. Any product that may be evaluated in this article, or claim that may be made by its manufacturer, is not guaranteed or endorsed by the publisher.
