# Supplementary material for: Human Multi-Compartment Airways-on-Chip Platform for Emulating Respiratory Airborne Transmission: From Nose to Pulmonary Acini
Source: Front Physiol. 2022 Mar 8;13:853317. doi: 10.3389/fphys.2022.853317 (PMC8957966; doi:10.3389/fphys.2022.853317)
Supplement: Supplementary file 2 [file Data_Sheet_2.pdf]

frontiers

Supplementary Material

1 Supplementary Data

Supplementary Material should be uploaded separately on submission. Please include any supplementary data, figures and/or tables. All supplementary files are deposited to FigShare for permanent storage and receive a DOI.

Supplementary material is not typeset so please ensure that all information is clearly presented, the appropriate caption is included in the file and not in the manuscript, and that the style conforms to the rest of the article. To avoid discrepancies between the published article and the supplementary material, please do not add the title, author list, affiliations or correspondence in the supplementary files.

2 Supplementary Figures and Tables

For more information on Supplementary Material and for details on the different file types accepted, please see here. Figures, tables, and images will be published under a Creative Commons CC-BY licence and permission must be obtained for use of copyrighted material from other sources (including re-published/adapted/modified/partial figures and images from the internet). It is the responsibility of the authors to acquire the licenses, to follow any citation instructions requested by third-party rights holders, and cover any supplementary charges.

2.1 Supplementary Figures

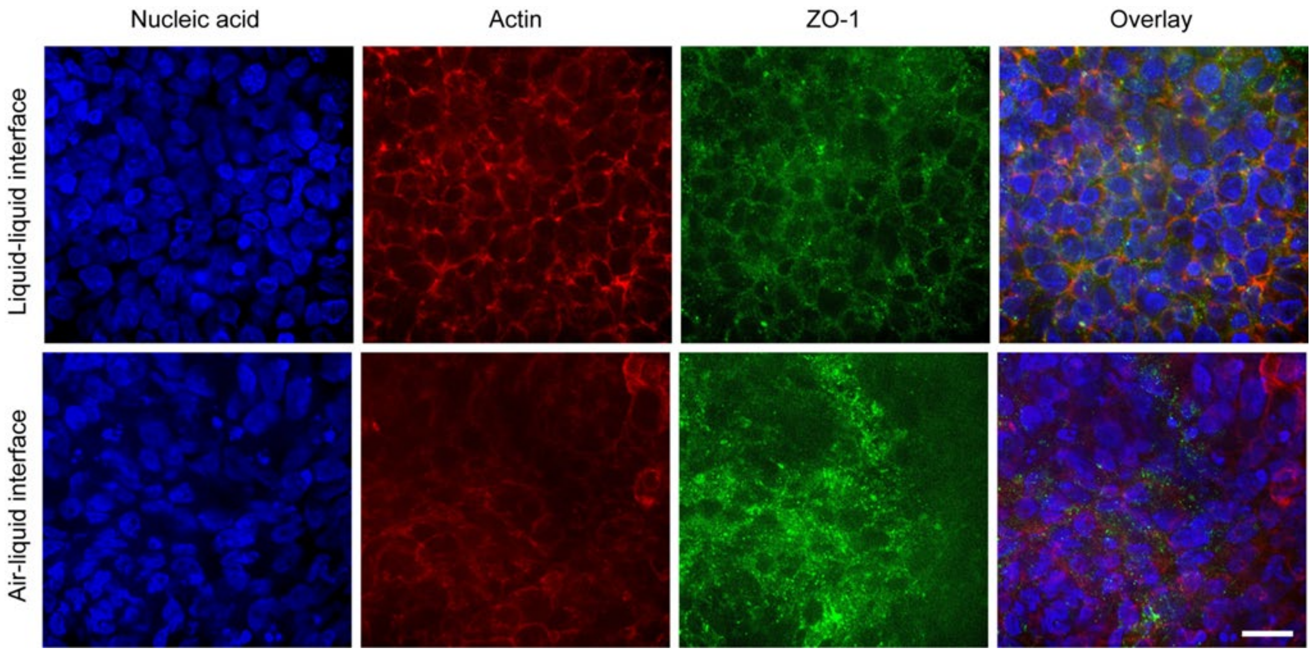

Supplementary Figure 1. Characterization of RPMI-2650 cells in 24 PET inserts. Top row shows cells grown in liquid-liquid interface (LLI) conditions stained for nucleic acid, actin, ZO-1, and an overlay (from left). Bottom row shows cells grown under air-liquid interface (ALI) conditions. Scale bar is 20 μm for all images.

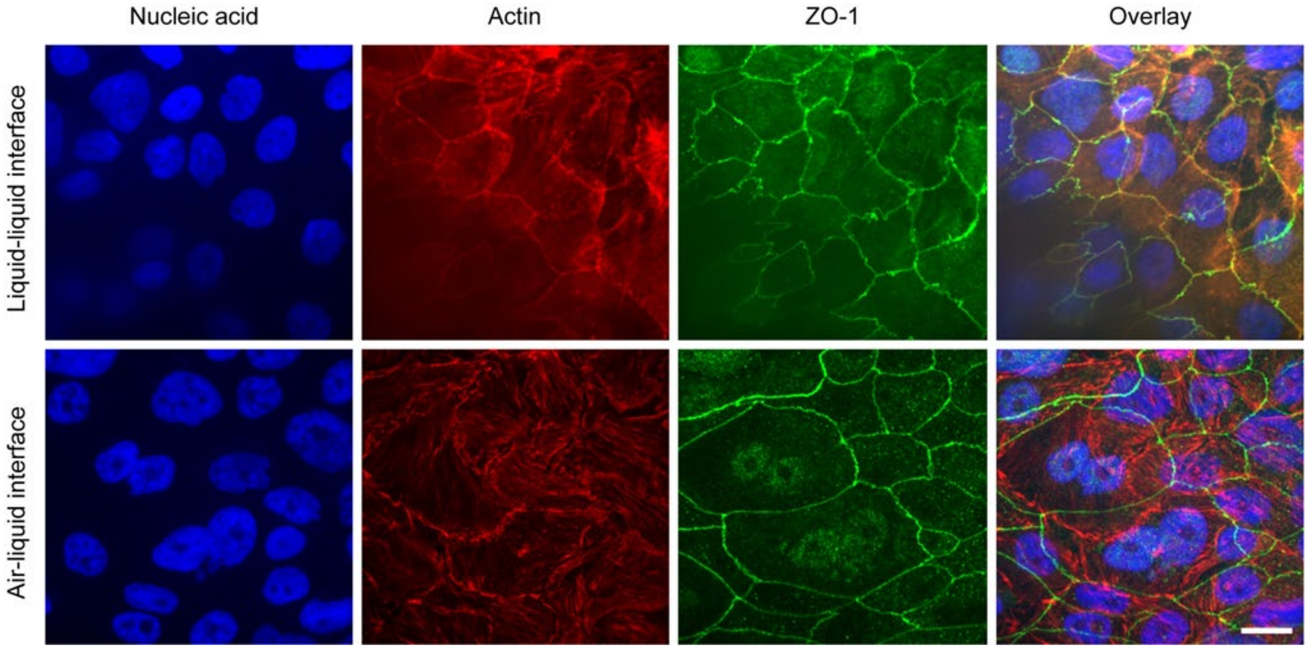

Supplementary Figure 2. Characterization of Calu-3 cells in 24 PET inserts. Top row shows cells grown in liquid-liquid interface (LLI) conditions stained for nucleic acid, actin, ZO-1, and an overlay (from left). Bottom row shows cells grown under air-liquid interface (ALI) conditions. Scale bar is 20 μm for all images.

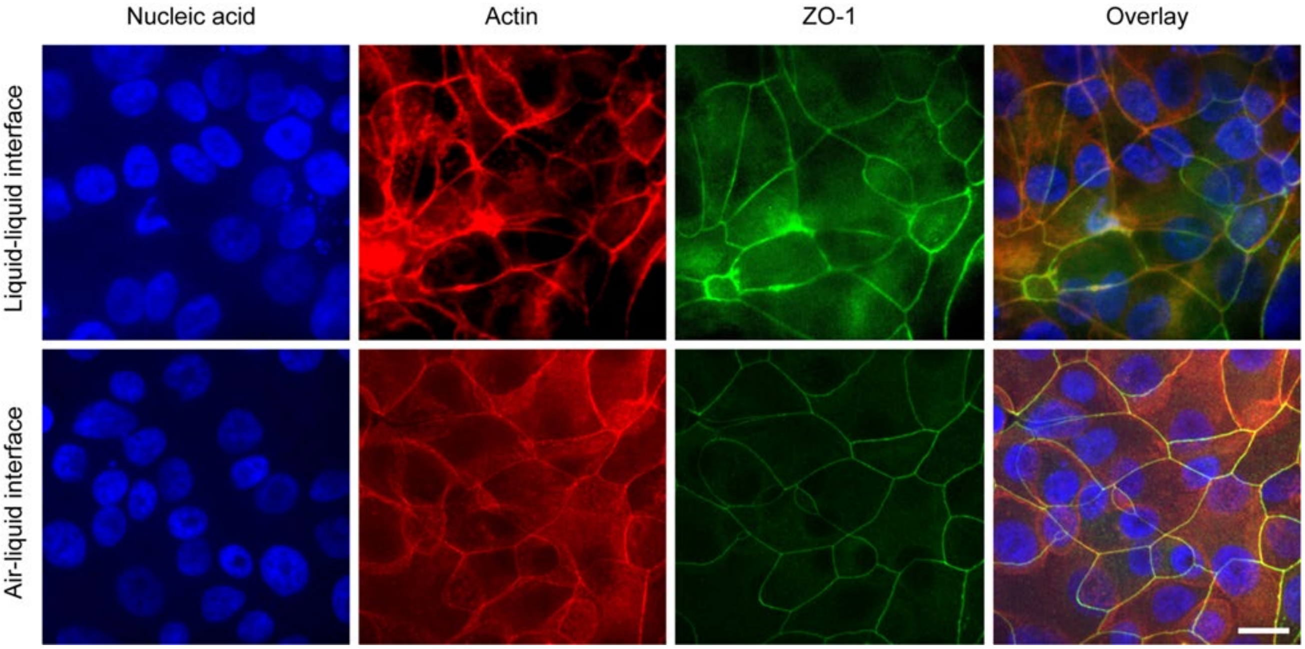

Supplementary Figure 3. Characterization of hAELVi cells in 24 PET inserts. Top row shows cells grown in liquid-liquid interface (LLI) conditions stained for nucleic acid, actin, ZO-1 and an overlay (from left). Bottom row shows cells grown under air-liquid interface (ALI) conditions. Scale bar is 20 µm for all images.

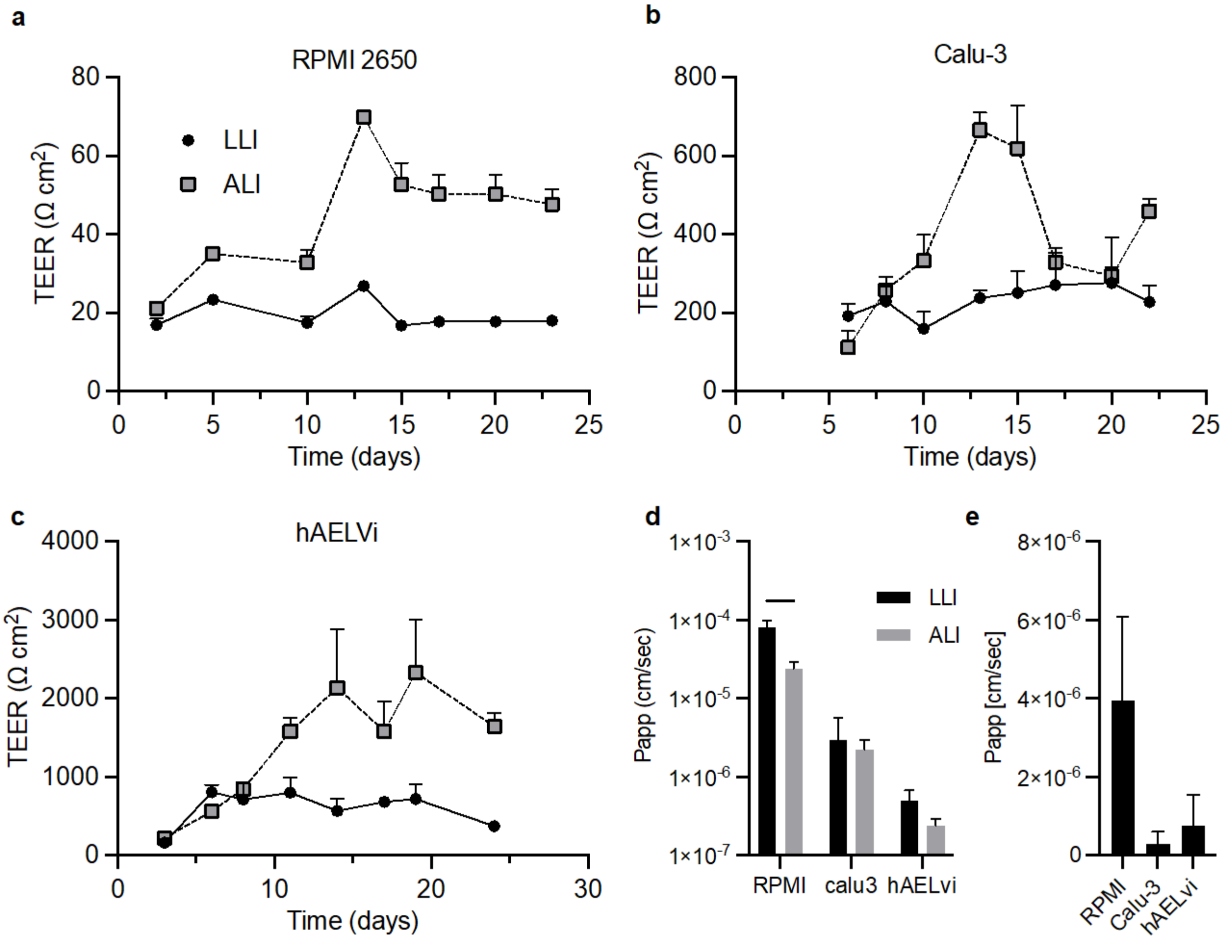

Supplementary Figure 4. Membrane integrity studies of the three cell-line monolayers: **(a)** RPMI-2650, **(b)** Calu-3, and **(c)** hAELVi. Transepithelial electrical resistance (TEER) is measured in 24 transwells under liquid- and air-liquid interface conditions (LLI and ALI, respectively) for a period of ~three weeks. All membranes exhibit higher trans-epithelial electrical resistance (TEER) following culture in ALI, relative to LLI with peak TEER following approximately 2-3 weeks of incubation. **(d)** Fluorescein sodium salt (FluNa) transport assay, a functional permeability test for cellularized models, was conducted in cell-seeded transwells by 3 weeks of culture. Apparent permeability coefficients (Papp) are higher under LLI conditions, further demonstrating the poorer membrane integrity relative to ALI conditions. Between the three cell populations, a monotonically decreasing membrane integrity trend is observed with the highest membrane integrity at the deep acinar region (hAELVi cells), the lowest at the most distal anatomic region represented by the RPMI 2650 cell line and intermediate values occupied by the calu-3 cells representing the bronchial region that is situated anatomically between these two extremes. **(e)** Apparent permeability coefficients (Papp) in cell line seeded models following three weeks in LLI growth conditions.

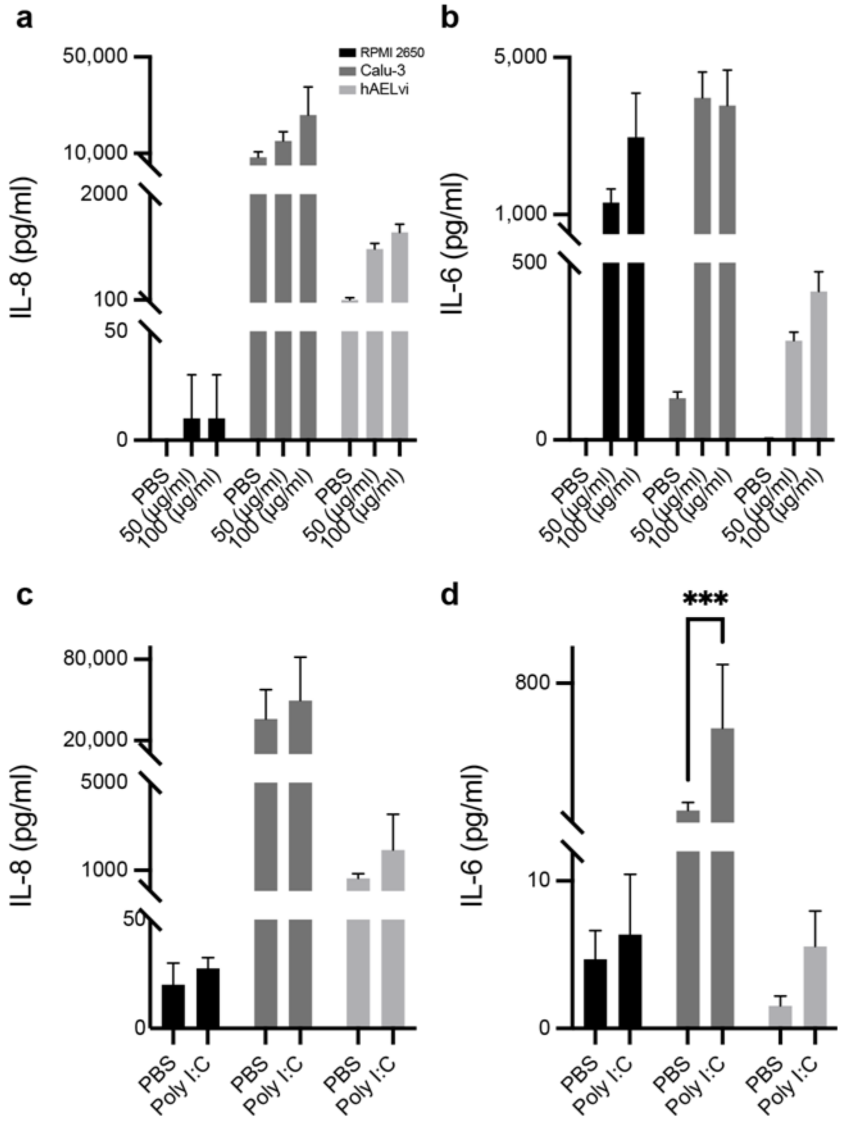

Supplementary Figure 5. Cytokine measurements in sampled medium from each of the model's three compartments following Poly(I:C) exposure. (a) and (b) plot the secretion of IL-6 and IL-8, respectively, under calibration conditions (transwell) while (c) and (d) plot IL-6 and IL-8 following direct exposure to Poly(I:C).
